# The complete mitochondrial genome of the *Schizothorax curilabiatus* (Cypriniformes: Cyprinidae)

**DOI:** 10.1080/23802359.2017.1383202

**Published:** 2017-09-27

**Authors:** Wanliang Wang, Liheng Zhang, Yingzi Pan, Tashi Lhamo, Chi Zhang, Baohai Li

**Affiliations:** a Fisheries Research Institute, Tibet Academy of Agricultural and Animal Husbandry Sciences, Lhasa, Tibet, China;; b Henan University of Animal Husbandry and Economy, Zhengzhou, China

**Keywords:** *Schizothorax curilabiatus*, mitochondrial genome, phylogenetic

## Abstract

The complete mitochondrial DNA sequence of *Schizothorax curilabiatus* was determined and analyzed. This mitochondrial genome was 16,578bp in length and showed significant AT bias (55.5% AT content, 44.5% GC content). Phylogenetic analysis showed that *S. curilabiatus* is more closely related to S. *oconnori* than to other species. The complete mitochondrial genome sequence provides an important dataset for the exploration of mitochondrial inheritance mechanism.

*Schizothorax curilabiatus* categorized into subfamily Schizothoracinae, family Cyprinidae, order Cypriniformes. It is only distributed in MoTuo reach of the Yarlung Zangbo River, southwest China (Wu and Wu 1992). This place has an exceptional aquatic ecosystem, unique geology, topography and climate. Till now, little is known about the mitochondrial genome information of this species. Here, we reported the complete mitogenome sequence of *S. curilabiatus*, which can be used in the studies on molecular systematics, phylogeography, stock evaluation, and conservation genetics.

The sample of *S. curilabiatus* was obtained from MoTuo (29°15′–29°21′N; 95°11′–95°22′E) at an altitude of 900 m in Tibet, China. A small portion of right pelvic fin was excised and preserved in the Fisheries Research Institute, Tibet Academy of Agricultural and Animal Husbandry Sciences. The total genomic DNA was extracted from the pelvic fin preserved in 95% alcohol. According to the mtDNA sequence of *Schizothorax waltoni* (Accession No. KC513574) (Chen et al. 2013), primers were designed for PCR amplification and sequencing.

The mitogenome of *S. curilabiatus* was a circular molecule of 16,578bp in length (Accession No MF804977).It consists of 13 protein-coding genes, 2 rRNAs, 22 tRNAs, and 2 non-coding regions, which again shows a typical gene arrangement of vertebrate mitogenomes (Anderson et al. 1981). Overall base composition of *S. curilabiatus*’s mitogenome is 30.1% for A, 27.0% for C, 17.5% for G, and 25.4% for T, with a high A + T content (55.50%), indicating an obvious anti-guanine bias commonly observed in teleost fishes (Jondeung et al. 2007).

As showed in neighbour-joining tree (by MEGA7.0 US) based on complete mitochondrial genomes of 17 species and constructed with 1000 bootstrap replicates (Figure 1). The tree supports clear phylogenetic relationships at the genus level. The complete mitochondrial genome sequence of *S. curilabiatus* provides an important data set for further study in mitochondrial inheritance mechanism.

**Figure 1.**
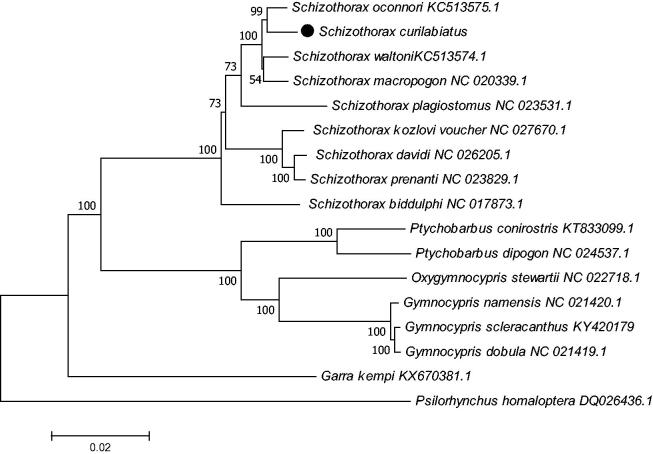
A neighbour-joining (NJ) tree of the *G. scleracanthus* was constructed using mitogenome sequences. The phylogenic tree is constructed by maximum-likelihood method with 1000 bootstrap replicates. GenBank accession numbers of mitogenomic sequences for each taxon are shown in parentheses.
